# LuxS-dependent AI-2 production is not involved in global regulation of natural product biosynthesis in *Photorhabdus* and *Xenorhabdus*

**DOI:** 10.7717/peerj.3471

**Published:** 2017-06-26

**Authors:** Antje K. Heinrich, Merle Hirschmann, Nick Neubacher, Helge B. Bode

**Affiliations:** 1 Fachbereich Biowissenschaften, Merck Stiftungsprofessur für Molekulare Biotechnologie, Goethe-Universität Frankfurt, Frankfurt am Main, Germany; 2 Buchmann Institute for Molecular Life Sciences, Goethe-Universität Frankfurt, Frankfurt am Main, Germany

**Keywords:** LuxS, Autoinducer-2, *Photorhabdus*, *Xenorhabdus*, Natural products, Regulation, Secondary metabolism, Quorum sensing

## Abstract

The Gram-negative bacteria *Photorhabdus* and *Xenorhabdus* are known to produce a variety of different natural products (NP). These compounds play different roles since the bacteria live in symbiosis with nematodes and are pathogenic to insect larvae in the soil. Thus, a fine tuned regulatory system controlling NP biosynthesis is indispensable. Global regulators such as Hfq, Lrp, LeuO and HexA have been shown to influence NP production of *Photorhabdus* and *Xenorhabdus*. Additionally, photopyrones as quorum sensing (QS) signals were demonstrated to be involved in the regulation of NP production in *Photorhabdus.* In this study, we investigated the role of another possible QS signal, autoinducer-2 (AI-2), in regulation of NP production. The AI-2 synthase (LuxS) is widely distributed within the bacterial kingdom and has a dual role as a part of the activated methyl cycle pathway, as well as being responsible for AI-2 precursor production. We deleted *luxS* in three different entomopathogenic bacteria and compared NP levels in the mutant strains to the wild type (WT) but observed no difference to the WT strains. Furthermore, the absence of the small regulatory RNA *micA*, which is encoded directly upstream of *luxS*, did not influence NP levels. Phenotypic differences between the *P. luminescens luxS* deletion mutant and an earlier described *luxS* deficient strain of *P. luminescens* suggested that two phenotypically different strains have evolved in different laboratories.

## Introduction

*Photorhabdus* and *Xenorhabdus* belong to the class of entomopathogenic bacteria that are able to infect and kill insects (Goodrich-Blair & Clarke, 2007). In nature, they live in symbiosis with nematodes of the family *Heterorhabditis* or *Steinernema,* respectively, and together they infect insect larvae. As symbionts, the bacteria supply compounds that support the nematode host development, but also toxic natural products (NP) and proteins that kill the insect prey (Bode, 2009). It is easy to imagine that in the complex life style of these bacteria, inter- (bacteria-nematode, bacteria-insect, bacteria-bacteria (food predators)) and intraspecies signaling or communication plays an important role. Signal molecule dependent communication in bacteria is referred to as “quorum sensing” (QS). While in Gram-negative bacteria QS often relies on acyl homoserine lactones (AHL), summarized under the term autoinducer-1 (AI-1) (Miller & Bassler, 2001), chemically different QS molecules binding to LuxR solos, have been identified in *Photorhabdus* (Brachmann et al., 2013; Brameyer et al., 2015). In contrast to the QS systems of Gram-negative bacteria, Gram-positive bacteria often use modified oligopeptides as QS signals (Waters & Bassler, 2005).

With the discovery of AI-2 and its corresponding synthase, LuxS, the first possible interspecies QS system was found, as the synthase is widespread among the bacterial kingdom in Gram-positive and -negative bacteria (Pereira, Thompson & Xavier, 2013). The reason for this frequent occurrence is the enzymatic role of LuxS in the activated methyl cycle (AMC) of some bacteria in which *S*-adenosylhomocysteine (SAH) is recycled to recover *S*-adenosylmethionine (SAM) (Pereira, Thompson & Xavier, 2013). During this cycle SAH is converted to homocysteine either by a one-step reaction using the enzyme SAH hydrolase (SahH) or a two-step reaction that requires the SAH nucleosidase (Pfs) and LuxS (Winzer et al., 2002). Pfs converts SAH to S-ribosylhomocysteine (SRH), which is further transformed to homocysteine by LuxS. A “by-product” of this reaction is 4,5-dihydroxy-2,3-pentanedione (DPD), which can rearrange to *R*- or *S*-2-methyl-2,3,3,4-tetrahydroxytetrahydrofuran (*R*- or *S*-THMF), both better known as AI-2. *S*-THMF-borate binds to the AI-2 sensor LuxP of *Vibrio harveyi,* while the AI-2 receptor LsrB of *Salomonella typhimurium* binds the borate-free form *R*-THMF (Chen et al., 2002; Miller et al., 2004). Therefore, two distinct AI-2 forms are bound by two different AI-2 receptors, the first system being unique to the *Vibrionaceae*. The Lsr transporter (*luxS* regulated) is encoded by eight genes (*lsrABCDFGKR),* which are arranged in two operons (Taga, Miller & Bassler, 2003). As described, LsrB is the receptor for AI-2 that is then transported through the outer membrane via the membrane channel formed by LsrCD into the cell (Rezzonico, Smits & Duffy, 2012). Energy for this process is provided by the ATPase, LsrA. The kinase, LsrK, phosphorylates AI-2 in the cytoplasm and the phosphorylated AI-2 activates the transcription of the *lsr* operon by releasing the repressor, LsrR.

By generating *luxS* mutants in bacterial strains, diverse phenotypes were attributed to QS by AI-2 (Rezzonico & Duffy, 2008). With the finding that LuxS is not exclusively an AI-2 synthase, it became clear that one has to be careful when analyzing *luxS* mutants, not confusing metabolic effects with real QS-related phenotypes. Beside the previously mentioned LuxR solos, *P. luminescens* TT01 and *Xenorhabdus* strains also encode the AI-2 synthase LuxS in their genomes (Duchaud et al., 2003). For *P. luminescens,* a *luxS* mutant was generated and phenotypically investigated by Krin et al. (2006). Interestingly, beneath phenotypic differences in bioluminescence, oxidative stress resistance, biofilm formation, virulence and twitching motility, the *luxS* deficient strain showed altered carbapenem-like antibiotic production (Derzelle et al., 2002) and altered expression of a non-ribosomal peptide synthetase (NRPS) gene cluster with a yet unknown NP (Krin et al., 2006). Recently it became clear that global regulators or QS signals can alter the production of NPs in *Photorhabdus/Xenorhabdus* (Brameyer et al., 2014). Hfq was identified as a regulator of various NPs in *P. luminescens* (Tobias et al., 2017), as well as LeuO, HexA and Lrp in *P. luminescens*, *X. nematophila* and *X. szentirmaii* (Engel et al., 2017). In order to investigate if LuxS plays a similar role in NP regulation in these three strains, the respective *luxS* deletion strains were constructed and analyzed for NP production.

## Material & Methods

### Bacterial cultivation

*Photorhabdus* and *Xenorhabdus* strains were cultivated in LB broth (10 g/l tryptone, 5 g/l yeast extract and 5 g/l NaCl) or Schneider’s insect medium (Sigma Aldrich, St. Louis, MO, USA) with constant shaking (200 rpm unless otherwise stated) at 30 °C. All *E. coli* strains were grown in LB broth with shaking at 37 °C. For plate cultures LB medium contained 1.5% agar. Chloramphenicol (34 µg/ml) was added to the medium when cultivated strains carried a plasmid. During conjugation of *P. luminescens* and *X. nematophila* using *E. coli* S17 *λ*pir, rifampicin (50 µg/ml) was used for selection against *E. coli*. When a plasmid was transferred into *X. szentirmaii* via conjugation ampicillin (100 µg/ml) was used for the same purpose. To enable growth of *E. coli* ST18, media were supplemented with 50 µg/ml *δ*-aminolevulinic acid (ALA). All strains used in this study are listed in Table 1.

**Table 1:** Bacterial strains used in this study.

Strain	Description/Genotype	Reference/source
*E. coli*		
DH10B	F^−^*araDJ39*Δ(*ara*, *leu*)7697 Δ*lacX74 galU galK rpsL deoRϕ*8O*dlac*ZΔM15 *endA*I *nupG recAl mcrA*Δ(*mrr hsdRMS mcrBC*)	Grant et al. (1990), Durfee et al. (2008)
S17 λpir	Tp Sm^r^*recA* thi hsdRM^+^ RP4::2-Tc::Mu::Km Tn7, λpir phage lysogen	Simon, Priefer & Pühler (1983)
ST18	S17 λpir Δ*hemA*	Thoma & Schobert (2009)
*Photorhabdus luminescens* TT01		
*P. luminescens*^G^	WT, rif^R^ (spontaneous)	Fischer-Le Saux et al. (1999), Bennett & Clarke (2005)
*P. luminescens*Δ*luxS*^G^	Deletion of *luxS* in *P. luminescens*^G^	This study
*P. luminescens*Δ*micA*^G^	Deletion of *micA* in *P. luminescens*^G^	This study
*P. luminescens*^F^	WT	Fischer-Le Saux et al. (1999)
*P. luminescens luxS*::*cm*^F^	Deletion of *luxS* and insertion of a chloramphenicol resistance cassette	Derzelle et al. (2002)
*Xenorhabdus szentirmaii* DSM 16338		
*X. szentirmaii*	WT	Gualtieri et al. (2014)
*X. szentirmaii*Δ*luxS*	Deletion of *luxS* in *X. szentirmaii*	This study
*Xenorhabdus nematophila* HGB081		
*X. nematophila*	WT, rif^R^ (spontaneous)	Orchard & Goodrich-Blair (2004)
*X. nematophila*Δ*luxS*	Deletion of *luxS* in *X. nematophila*	This study
*Enterobacter hormaechei*		
ATCC 700323		ATCC^®^
*Enterobacter cloacae*		
NEG 03 51713981	Clinical isolate	
NEG 80 51755054	Clinical isolate	

**Notes.**

The superscripted letters G and F differentiate between strains which were derived from the *P. luminescens* TT01 WT strain which is used in Germany in the Bode laboratory (G) and the P. *luminescens* TT01 strains which were used in France (F) (Krin et al., 2006), and were kindly provided by Evelyne Krin. All *Enterobacter* strains were kindly provided by Thomas A. Wichelhaus.

**Table 2:** Oligonucleotides used in this study.

Name	Sequence (5′ → 3′)	Purpose
Δ*plu1253*_up_PstI-Gib_fw	CCTCTAGAGTCGACCTGCAGTGACGA GTTTGCTAAATTGG	Amplification up- and downstream product for the deletion of *luxS* (*plu1253*) in *P*. *luminescens*
Δ*plu1253*_up_Gib_rev	ACTACTATGGAACAAAAAATTCAGAT TTTTCTTCAAG	
Δ*plu1253*_do_Gib_fw	CTGAATTTTTTGTTCCATAGTAGTGAT AATATTTCGG	
Δ*plu1253*_do_BglII-Gib_rev	TCCCGGGAGAGCTCAGATCTCCCGTA ATGAAATTGTTGG	
Δ*luxS*__TT01_mut_ver_fw	AGATGGAACTTGTTATCTGCC	Verification of Δ*plu1253*
Δ*luxS*__TT01_mut_ver_rev	AGTTATGCCAAAAACGATAGC	
Δ*XNC1_1265*_up_PstI_Gib_fw	CCTCTAGAGTCGACCTGCAGAAGCAA TTTGTAAACCGTCC	Amplification up- and downstream product for the deletion of *luxS* (*XNC1_1265*) in *X. nematophila*
Δ*XNC1_1265*_up_Gib_rev	CTAAATACACAGATACATTACCTCCTA AGGTATCAGATT	
Δ*XNC1_1265*_do_Gib_fw	AGGAGGTAATGTATCTGTGTATTTAGC GGTTATCG	
Δ*XNC1_1265*_do_BglII-Gib_rev	TCCCGGGAGAGCTCAGATCTAATCAA ACACCAATCTATCACG	
Δ*luxS*__*XNC1* _mut_ver_fw	TCTGTTCTTCATTCTTACGAGG	Verification of Δ*XNC1_1265*
Δ*luxS*__*XNC1* _mut_ver_rev	ATTGTTCATGCGTTGTATAGG	
ΔXSZ_*luxS*_up_PstI_Gib_fw	CCTCTAGAGTCGACCTGCAGCTTCAGA TGCTTTGTTACGAGG	Amplification up- and downstream product for the deletion of *luxS* (*XSR1_140025)* in *X. szentirmaii*
ΔXSZ_*luxS*_up_Gib_rev	ATGCCAATTCCGCCATTCGTGTATGGTC	
ΔXSZ_*luxS*_do_Gib_fw	ACGAATGGCGGAATTGGCATTGCCTGAAG	
ΔXSZ_*luxS*_do_BglII-Gib_rev	TCCCGGGAGAGCTCAGATCTTTAACA CATTCTCCGCATGG	
Δ*luxS*__XSZ_mut_ver_fw	GACTTGCTATTTGCCTTATGC	Verification of Δ*XSR1_140025*
Δ*luxS*__XSZ_mut_ver_rev	TCTCGAGAAAGTGACTGTCG	
Δ*micA*_TT01_up_fw	TCGATCCTCTAGAGTCGACCTGCAGCA CCAATAAATCACAGAGCG	Amplification up- and downstream product for the deletion of *micA* region (Papamichail & Delihas, 2006) in *P*. *luminescens*
Δ*micA*_TT01_up__rev	ACAAAAAATTCAGATTCTTTTCTAGCA TCCTGTCTG	
Δ*micA*_TT01_down_fw	ATGCTAGAAAAGAATCTGAATTTTTTG TGGAGATG	
Δ*micA*_TT01_down_rev	GGAATTCCCGGGAGAGCTCAGATCTG TATGGTGCTTGAAGAGTTGG	
V_ Δ*micA*_TT01_ii_fw	GGAAAAAATGAAGAGTCAGGG	Verification of Δ*micA*
V_Δ*micA*_TT01_ii_rev	TCTGCAACACGTACTTCTGC	
V_Δ*micA*_TT01_ai_fw	AGATGGAACTTGTTATCTGCC	Verification of Δ*micA*
V_Δ*micA*_TT01_ai_rev	AATTAAATAAAGCCTTCAACTGG	

**Notes.**

_fw forward primer _rev reverse primer

### Construction of *luxS* deletion strains

The deletion of the LuxS encoding gene (*plu1253*) in *P. luminescens* was realized by amplifying the up- and the downstream region of this gene using primers Δ*plu1253*_up_PstI-Gib_fw and Δ*plu1253*_up_Gib_rev, or Δ*plu1253*_do_Gib_fw and Δ*plu1253*_do_BglII-Gib_rev, respectively. All oligonucleotides that were used as primers are listed in Table 2. For the upstream region, a PCR product of 919 bp was generated and the downstream PCR product had a size of 795 bp. Both PCR products were fused and integrated into the *Pst*I and *Bgl*II linearized pCKcipB plasmid via Gibson cloning (Gibson Assembly^®^ Master Mix, New England Biolabs). To enable Gibson cloning, primers had homologous overhangs to either the up- or the downstream product or the vector. *E. coli* S17 λpir cells were transformed with the Gibson assembly using electroporation. Correctness of the constructed deletion plasmid pDelta_*plu1253* (Table 3) was confirmed after isolation via restriction digest and the plasmid was subsequently transferred into *P. luminescens* by conjugation. Conjugation of *P. luminescens* and chromosomal integration of the plasmid via a first homologous recombination as well as deletion of the gene of interest due to a second homologous recombination have been described previously (Brachmann et al., 2007). In order to differentiate between the desired deletion mutants and mutants genetically equal to the WT, the loss of the gene was confirmed via PCR with the primers Δ*luxS*__TT01_mut_ver_fw and Δ*luxS*__TT01_mut_ver_rev using chromosomal DNA as template. For the WT a 2,625 bp product was amplified, whereas the amplicon of the deletion mutant was only 2,096 bp long. The same strategy was used for construction of the plasmids pDelta_*XNC1_1265* and pDelta_*XSR1_140025* (Table 3) and the subsequent deletion of *luxS* in *X. nematophila* and *X. szentirmaii.* For *X. nematophila,* the up- and the downstream regions were amplified with the primers Δ*XNC1*_*1265_*up_PstI_Gib_fw/Δ*XNC1*_*1265*_ up_Gib_rev and Δ*XNC1*_*1265*_do_Gib_fw/Δ*XNC1*_*1265*_do_BglII-Gib_rev, yielding amplicons of 963 bp and 944 bp, respectively. The deletion of the gene was controlled with the primer pair Δ*luxS*__XNC1_mut_ver_fw and _rev (WT: 2626 bp and Δ*luxS* mutant: 2,110 bp). Upstream (857 bp) and downstream regions (793 bp) of *X. szentirmaii* were amplified (for this mutant only an internal 426 bp fragment of the gene was in-frame deleted) with ΔXSZ_*luxS*_up_PstI_Gib_fw/ΔXSZ_luxS_up_Gib_rev and ΔXSZ_*luxS*_do_Gib_fw/ΔXSZ_luxS_do_BglII-Gib_rev. The deletion was confirmed with primers Δ*luxS*__XSZ_mut_ver_fw and Δ*luxS*__XSZ_mut_ver_rev (WT: 2382 bp and Δ*luxS* mutant: 1,956 bp). Deletion of *micA* in *P. luminescens* was performed applying minor changes to the protocol described above. After Gibson cloning of the deletion plasmid, pDelta_*micA,* using the 884 bp upstream fragment (amplified with the primers Δ*micA*_TT01_up_fw and_rev), the 840 bp downstream fragment (amplified with the primers Δ*micA*_TT01_down_fw and _rev) and *Pst*I and *Bgl*II linearized pCKcipB plasmid in one assembly reaction, the assembly mixture was used to transform *E. coli* ST18 cells. For cultivation of *E. coli* ST18 cells, 50 µg/ml ALA was added to the media. Conjugation of the plasmid from ST18 cells to *P. luminescens*, chromosomal integration of the plasmid, excision of the plasmid via second homologous recombination and counter selection with sucrose were performed as described above. Deletion of *micA* was confirmed with primers V_Δ*micA*_TT01_ai_fw and _rev binding outside of the amplified region (WT: 1,879 bp and Δ*micA*: 1,760 bp). Due to the small size of the deleted region, additional verification primers, binding closer to the deleted region, were used. V_Δ*micA*_TT01_ii_fw and _rev leading to PCR products of 665 bp for the WT and 546 bp for Δ*micA.*

**Table 3:** Plasmids used in this study.

Plasmid	Description	Reference/source
pCKcipB	pDS132 (Philippe et al., 2004) based plasmid with an additional *Bgl*II restriction site, R6K ori; cm^R^; oriT; *sacB*; relaxase *traI*	Nollmann et al. (2015)
pDelta_*plu1253*	pCKcipB based deletion plasmid encoding fused *plu1253* up- (919 bp) and downstream (795 bp) regions	This study
pDelta_*XNC1_1265*	pCKcipB based deletion plasmid encoding fused *XNC1_1265* up- (963 bp) and downstream (944 bp) regions	This study
pDelta_*XSR1_140025*	pCKcipB based deletion plasmid encoding fused *XSR1_140025* up- (857 bp) and downstream (793 bp) regions	This study
pDelta_*micA*	pCKcipB based deletion plasmid encoding fused *micA* up- (884 bp) and downstream (840 bp) regions	This study

### Bioinformatic analysis

The *luxS* gene and the *lsr* operon in *P. luminescens subsp. laumondii* TT01 (NC_005126.1), *X. nematophila* ATCC 19061 (NC_014228.1) and *X. szentirmaii* DSM 16338 (NZ_CBXF000000000.1) were identified by a tblastn (Basic Local Alignment Search Tool, NCBI) search. LuxS and the Lsr proteins of *E. coli* K-12 were used as queries (Accession numbers: LuxS: CQR82138.1, LsrKRACDBFG: CQR81040.1–CQR81047.1).

### NP quantification

In order to compare NP production, analytical culture extracts were prepared. 10 ml LB medium with or without 2% of Amberlite^®^ XAD-16 (Sigma-Aldrich, St. Louis, MO, USA) (XAD) were inoculated with a starting OD_600_ = 0.1 using an overnight culture. After 72 h of cultivation at 30 °C either XAD or ethyl acetate (EE) culture extracts were prepared as described before (Nollmann et al., 2015; Heinrich et al., 2016). XAD extracts of *P. luminescens* TT01 WT and Δ*luxS* were prepared after 48 h of cultivation. Briefly, XAD was separated from the supernatant and extracted with methanol (MeOH). After filtration, the crude extract was dried under reduced pressure. For HPLC-UV/MS analysis, extracts were dissolved in one culture volume of MeOH. For EE extracts 2 ml culture was extracted with an equal volume of EE. After phase separation 1 ml of the EE phase was dried under nitrogen flow and dissolved in 250 µl of MeOH. XAD extracts were prepared in quintuplicates and EE extract in quadruplicates. For this, five (XAD) or four (EE) individual cultures were inoculated with the same overnight culture and used for extraction. HPLC-UV/MS analysis was done as previously stated (Reimer et al., 2011). A total of 5 µl of each sample was separated on a C_18_-UHPLC column (Acquity UPLC BEH C18 1.7 lmRP 2.1 × 50 mm (Waters)) with a C_18_-pre-column (Acquity UPLC BEH C18 1.7 lmRP 2.1 × 5 mm (Waters)) using a H_2_O in acetonitrile (ACN) gradient. Both solvents were supplemented with 0.1% formic acid (FA). The gradient was either from 5–95% (ACN) in 16 min with a flow rate of 0.4 ml/min and 40 °C (XAD extracts) or from 5–95% in 22 min with 0.6 ml/min at 30 °C (EE extracts). Relative quantification of the NPs was performed as explained previously (Heinrich et al., 2016) using the software Bruker Compass DataAnalysis 4.3 for HPLC-MS data analysis and TargetAnalysis Version 1.3 for quantification of the peak area of the different compounds. The *m*∕*z* ratios which were used for generation of extracted ion chromatograms (EICs) for the quantification of the respective compounds are listed in Table 4.

**Table 4:** Identified and quantified compounds.

Name	Abbreviation	*m*∕*z*	Ion	Reference
Isopropylstilbene	IPS	255.1	[M+H]^+^	Joyce et al. (2008)
Anthraquinone 284	AQ-284	285.1	[M+H]^+^	Brachmann et al. (2007)
Anthraquinone 270a	AQ-270a	271.1	[M+H]^+^	Brachmann et al. (2007)
GameXPeptide A	GXP-A	586.4	[M+H]^+^	Bode et al. (2012)
GameXPeptide B	GXP-B	600.4	[M+H]^+^	Bode et al. (2012)
GameXPeptide C	GXP-C	552.4	[M+H]^+^	Bode et al. (2012)
Photopyrone C	PPY-C	281.2	[M+H]^+^	Brachmann et al. (2013)
Photopyrone D	PPY-D	295.2	[M+H]^+^	Brachmann et al. (2013)
Photopyrone E	PPY-E	309.2	[M+H]^+^	Brachmann et al. (2013)
Photopyrone F	PPY-F	323.3	[M+H]^+^	Brachmann et al. (2013)
Desmethyl phurealipid A	dmPL-A	215.2	[M+H]^+^	Nollmann et al. (2015)
Phurealipid A	PL-A	229.2	[M+H]^+^	Nollmann et al. (2015)
Phurealipid C	PL-C	243.2	[M+H]^+^	Nollmann et al. (2015)
Phurealipid B	PL-B	257.3	[M+H]^+^	Nollmann et al. (2015)
Mevalagmapeptide	MVAP	334.8	[M+2H]^++^	Bode et al. (2012)
Nematophin	NMT	273.2	[M+H]^+^	Cai et al. (2017a), Li, Chen & Webster (1997)
Rhabdopeptide 1	RXP-1	574.4	[M+H]^+^	Reimer et al. (2013)
Rhabdopeptide 2	RXP-2	588.4	[M+H]^+^	Reimer et al. (2013)
Rhabdopeptide 3	RXP-3	687.5	[M+H]^+^	Reimer et al. (2013)
Rhabdopeptide 4	RXP-4	701.5	[M+H]^+^	Reimer et al. (2013)
Rhabdopeptide 5	RXP-5	800.6	[M+H]^+^	Reimer et al. (2013)
Rhabdopeptide 6	RXP-6	814.6	[M+H]^+^	Reimer et al. (2013)
Xenematide A	XMT-A	663.3	[M+H]^+^	Lang et al. (2008)
Xenocoumacine I	XNC-I	466.3	[M+H]^+^	McInerney et al. (1991), Reimer et al. (2009)
Xenocoumacine II	XNC-II	407.2	[M+H]^+^	McInerney et al. (1991), Reimer et al. (2009)
Xenocoumacine III	XNC-III	405.2	[M+H]^+^	Reimer et al. (2009)
Xenortide A	XP-A	410.3	[M+H]^+^	Lang et al. (2008)
Xenortide B	XP-B	449.3	[M+H]^+^	Lang et al. (2008)
Xenotetrapeptid	XTP	411.3	[M+H]^+^	Kegler et al. (2014)
Szentiamide	SZT	838.4	[M+H]^+^	Ohlendorf et al. (2011), Nollmann et al. (2012)
Xenofuranon A	XF-A	281.1	[M+H]^+^	Brachmann et al. (2006)
Xenoamicin A	XAB-A	650.9	[M+2H]^2+^	Zhou et al. (2013)
Xenoamicin B	XAB-B	657.9	[M+2H]^2+^	Zhou et al. (2013)
Rhabdopeptide 771	RXP-771	772.6	[M+H]^+^	Cai et al. (2017b)
Rhabdopeptide 884	RXP-884	885.6	[M+H]^+^	Cai et al. (2017b)

### Carbapenem production assay

The carbapenem plate assay was performed as described earlier (Derzelle et al., 2002). Agar plates with 72 h old spots of *P. luminescens* WT^G^, Δ*luxS*^G^, Δ*micA*^G^, WT^F^ and *luxS*::*cm*^F^ were overlaid with swarm agar (0.6%) (sifin diagnostics gmbh) containing carbapenem sensitive *Enterobacter* strains (Table 1). The assay was performed for each strain in triplicates.

### Bioluminescence measurements

Precultures were grown over night in 10 ml LB medium inoculated from a single colony. From a preculture 10 ml of LB medium was inoculated with an OD_600_ = 0.1 in triplicates and cultivated at 30 °C. At defined time points, OD_600_ was measured and 100 µl of each culture were transferred into the well of a microtiter plate (corning 96 flat bottom white, clear bottom polystyrol, -pure Grade™ S-, Ref: 781,670, BRAND*plates*^®^). Bioluminescence was measured with an Infinite 200 PRO reader (Tecan Trading AG, Männedorf. Switzerland) (Shaking linear duration: 4 s, shaking linear amplitude: 1 mm, top reading, mode: luminescence, attenuation: none, integration time: 1,000 ms, settle time: 0 ms). For comparability, bioluminescence was normalized by division through the OD_600_ measured at the same time point. GraphPad Prism 7.00 (GraphPad Software, Inc, La Jolla, CA, USA) was used for calculating *P* values (unpaired *t*-test).

### Oxidative stress assay

Precultures were grown over night in 10 ml LB medium inoculated from a single colony. From a preculture, 40 ml of LB medium was inoculated with an OD_600_ = 0.25 and grown for ∼2 h until the culture had reached an OD_600_ = 0.5. 2 ml from the 40 ml culture was taken and H_2_O_2_ or paraquat was added in the tested concentrations, in triplicates. A total of 200 µl from the treated cultures were transferred into the well of a microtiter plate (Polystyrene (PS) Microtest Plate 96 Well.R, round bottom, Ref 82.1582.001, Sarstedt) and the OD_600_ was measured immediately in a SpectraMax 340PC384 Microplate Reader (SoftMax^®^ Pro; Molecular Devices, Sunnyvale, CA, USA). To avoid concentration differences due to water evaporation, the outer rows of the microtiter plate were left empty. The cultures were cultivated at 30 °C in the microtiter plate and the OD_600_ was measured every two hours.

### Biofilm assay

The ability to form biofilms on a plastic surface was monitored using a slightly modified version of the microtiter plate biofilm assay published by Merritt, Kadouri & O’Toole (2005). Cells of an overnight culture grown in LB medium were collected with centrifugation (2 min, 10,000× g, RT) and resuspended in Schneider’s insect medium adjusting to an OD_600_ = 0.6. For every strain, 100 µl was inoculated in six replicate wells (Polystyrene (PS) Microtest Plate 96 Well.R, round bottom, Ref 82.1582.001; Sarstedt, Nümbrecht, Germany) and incubated for 72 h at 30 °C in a humidified box. We cultivated the strains in Schneider’s insect medium, when performing the biofilm assay. The wells were washed twice with H_2_O and biofilms were stained with 0.1% crystal violet solution (solved in H_2_O) for 10 min. Unbound dye was removed and the stained biofilms were air dried. The amount of biofilm bound crystal violet serves as a measure for biofilm formation. Dye was dissolved using 30% acetic acid, with 100 µl of this solution transferred to a new microtiter plate (Polystyrene (PS) Microtest Plate 96 Well.F, flat bottom, Ref 82.1581.001; Sarstedt, Nümbrecht, Germany) for measuring the absorption at a wavelength of 570 nm using a microplate reader (Infinite 200 PRO reader; Tecan Trading AG, Männedorf. Switzerland).

### Virulence assay

Precultures were diluted to an OD_600_ = 0.3 in 10 ml LB broth and grown at 30 °C with shaking (200 rpm) to an OD_600_ = 1.2–1.5. After harvesting the cells using centrifugation (10,000× g, 1 min, RT), cell pellets were resuspended in LB_Tween_ (0.1% Tween80) and each cell suspension was serially diluted to a final OD_600_ = 0.0002. 15 *Galleria mellonella* larvae per strain were injected with 5 µl of diluted cell suspension. Larvae were incubated at 30 °C and the number of living individuals was monitored every hour. In order to compare the LT_50_ values, data are presented in a Kaplan–Meier curve using GraphPad Prism 7.00 (GraphPad Software, Inc, La Jolla, CA, USA).

### Determination of AI-2 in supernatants of *P. luminescens* cultures via GC-MS

The determination of AI-2 in the supernatants of *P. luminescens* was conducted in accordance with a protocol published previously (Thiel et al., 2009), which bases on the measurement of the precursor of AI-2, DPD. Briefly, every strain was inoculated in 10 ml of LB medium in triplicates from an overnight culture with an OD_600_ = 0.1 and cultivated for 24 h. LB medium without bacteria was used as a control. Cultures were centrifuged (4,000× g, 10 min, 4 °C) and 3 ml of the clear supernatant was transferred to a new 15 ml falcon and mixed with 1 ml derivatization reagent (0.1 M K_2_HPO_4_/KH_2_PO_4_ buffer, pH 7.2, supplemented with 50 mM *o*-phenylenediamine). After a 3 h incubation at RT (rolling), samples were extracted with 6 ml dichloromethane (DCM) and afterwards 4 ml of the organic (lower) phase were taken and dried under nitrogen flow. Dried samples were dissolved in 150 µl of DCM and 50 µl *N*-Methyl-(*N*-trimethylsilyl)-trifluoroacetamide (MSTFA) were added for derivatization. The reaction was carried out for 1 h at 60 °C. An Agilent gas chromatography (GC)-MS system with a 7890A gas chromatograph with a DB-5HT column (30 m by 250 µm by 0.1 µm) coupled to a 5975C mass spectrometer (scan range 40–300 m/z, EI ionization energy 70 eV) was used for analysis.

Two microliters of the sample were measured in split mode at a rate of 10:1. The helium flow rate was set to 1 ml/min. The inlet temperature was set to 300 °C. Analysis was performed with an initial oven temperature of 50 °C. The temperature was increased by 5 °C/min to 75 °C followed by 120 °C/min to 300 °C (hold for 5 min) and finally 120 °C/min to 50 °C (total runtime 15 min).

## Results

### Influence of *luxS* on NP production

**Figure 1:**
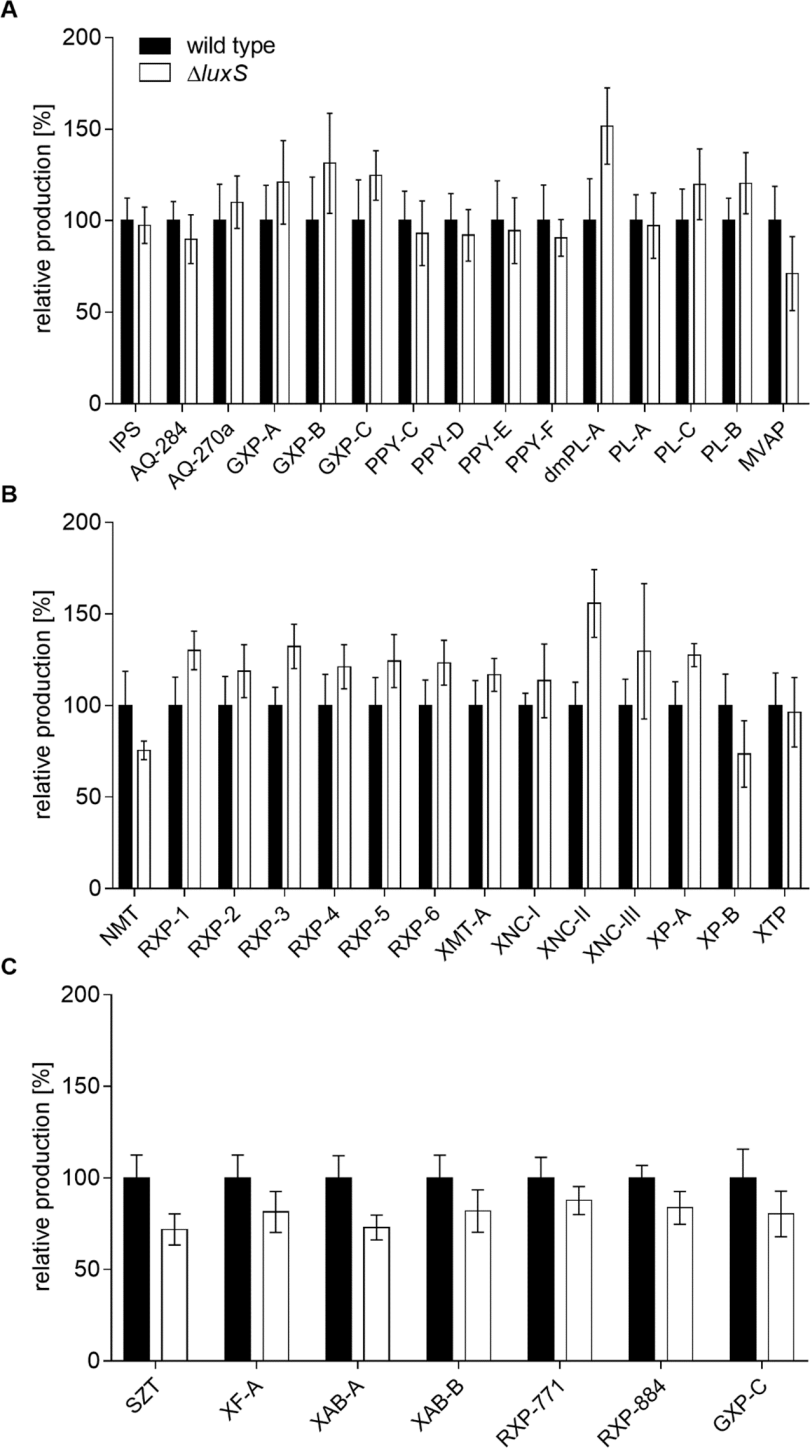
Comparison of NP production of wild type (black) and Δ*luxS* (white) strains of (A) *P. luminescens*^**G**^, (B) *X. szentirmaii* and (C) *X. nematophila*. Production was normalized using the OD_600_ when XAD extracts were prepared and is given relative to the wild type production of each compound. Experiments were performed in quintuplicates. For details see ‘Material and Methods’.

It was shown more than a decade ago that LuxS is involved in the regulation of carbapenem production in *P. luminescens* (Derzelle et al., 2002) and the role of AI-2-signaling in *P. luminescens* was revealed by transcriptome and proteome analysis (Krin et al., 2006). In this transcriptomic analysis, an NRPS encoding gene with a yet unknown product was upregulated in the absence of LuxS in the mid-exponential phase (Krin et al., 2006) even though it was suggested that it is difficult to see transcriptional changes of genes responsible for NP biosynthesis due to their (often) low transcriptional level. In an attempt to examine if secondary metabolism in *Photorhabdus* and *Xenorhabdus* in general is regulated by AI-2 controlled QS or by another LuxS dependent mechanism, homologs of *luxS* were deleted in three different entomopathogenic strains: *Photorhabdus luminescens*, *Xenorhabdus szentirmaii* and *Xenorhabdus nematophila*. These strains were chosen since their NP production had been studied in detail previously and several compounds with their corresponding biosynthetic genes are known. A first comparison between the Δ*luxS* mutants and the respective WT strains did not indicate any obvious phenotypic differences concerning colony morphology and growth behavior. Only colonies of the *P. luminescens* Δ*luxS* mutant older than seven days had a darker pigmentation than the WT colonies on LB agar plates. NP levels of extracts were compared by HPLC-MS analysis. Culture extracts of all investigated strains were prepared after cultivation in LB medium supplemented with XAD. None of the Δ*luxS* mutants showed a significantly altered NP production compared to the WT strains (Fig. 1, for NP abbreviations see Table 4).

**Figure 2:**
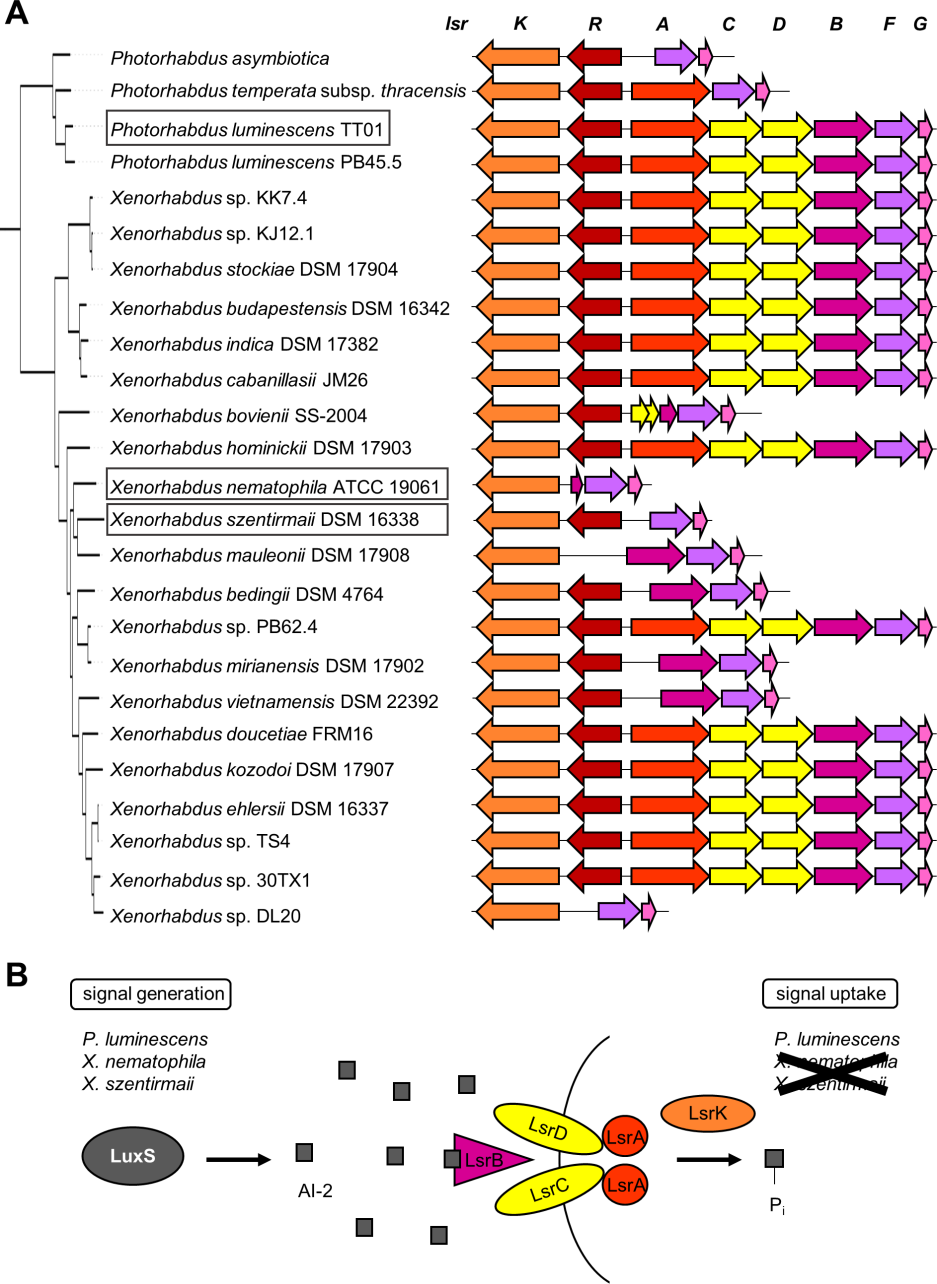
(A) Occurrence of the *lsr* operon in 25 analyzed *Xenorhabdus* and *Photorhabdus* strains. The phylogenetic tree is a trimmed version of an analysis described previously (Tobias NJ, Wolff H, Djahanschiri B, Grundmann F, Kronenwerth M, Shi Y-M, Simonyi S, Grün P, Shapiro-Ilan D, Pidot SJ, Stinear TP, Ebersberger I, Bode HB, 2017, unpublished data). LsrK: AI-2 kinase, LsrR: lsr operon transcriptional repressor, LsrA: AI-2 import ATP-binding protein, LsrC: AI-2 import system permease protein, LsrC: AI-2 import system permease protein, LsrB: AI-2 binding protein, LsrF: thiolase, LsrG: AI-2 degrading protein. (B) LuxS is required to build AI-2, so all strains investigated in detail in this study are able to generate a signal. When it comes to signal uptake only *P. luminescens* has all required genes for the internalization of AI-2.

### Presence of the AI-2 transporter genes *lsrABCDFGKR* in entomopathogenic bacteria

As no difference in the amount of the produced NPs between *P. luminescens*, *X. nematophila* and *X. szentirmaii* and their corresponding Δ*luxS* strains were detected, we decided to analyze which of these strains can use AI-2 as a signal molecule. The presence or the absence of the transporter (*lsr* operon) for AI-2 uptake in each strain indicates, whether AI-2 can act as a QS molecule or if it is nothing but a “by-product” of the LuxS catalyzed reaction in the AMC in the respective strain. When the complete *lsr* operon is present in the bacterial genome, it is reported that AI-2 can function as a signal molecule by this bacterium (Rezzonico & Duffy, 2008). The genomes of the three strains were examined for the presence of all Lsr proteins present in *E. coli* K-12 by a tblastn search. While the genome of *P. luminescens* harbors the complete set of *lsr* genes, the genomes of *X. nematophila* and *X. szentirmaii* encode only parts of it (Fig. 2A). *X. nematophila* encodes only *lsrKFG* and the 3′ end of *lsrB* and *X. szentirmaii* encodes *lsrKRFG*. A wider analysis of 25 *Xenorhabdus* and *Photorhabdus* strains revealed that the pattern of the *lsr* operon is not directly reflected by the phylogeny (Fig. 2A). Comparing the different structures of the *lsr* cluster with the phylogeny indicates that loss of *lsr* genes was not caused by an initial deletion event in one common ancestor, but is the result of several individual losses in the affected strains. Although ten strains have lost parts of the operon, the kinase LsrK and the AI-2 degrading proteins LsrFG are encoded in all strains investigated. LsrF has a thiolase activity and LsrG acts as an isomerase (Marques et al., 2014). We concluded from these results that in our selected strains, AI-2 can play a role as a signal molecule only in *P. luminescens* as the two other strains lack the important channel proteins for AI-2 transport across the outer membrane (Fig. 2B) even if other uptake mechanisms exist (see ‘Discussion’). Therefore, all following investigations concentrated on *P. luminescens*.

### Comparison of NP production of different *P. luminescens* WT and Δ*luxS* strains

**Figure 3:**
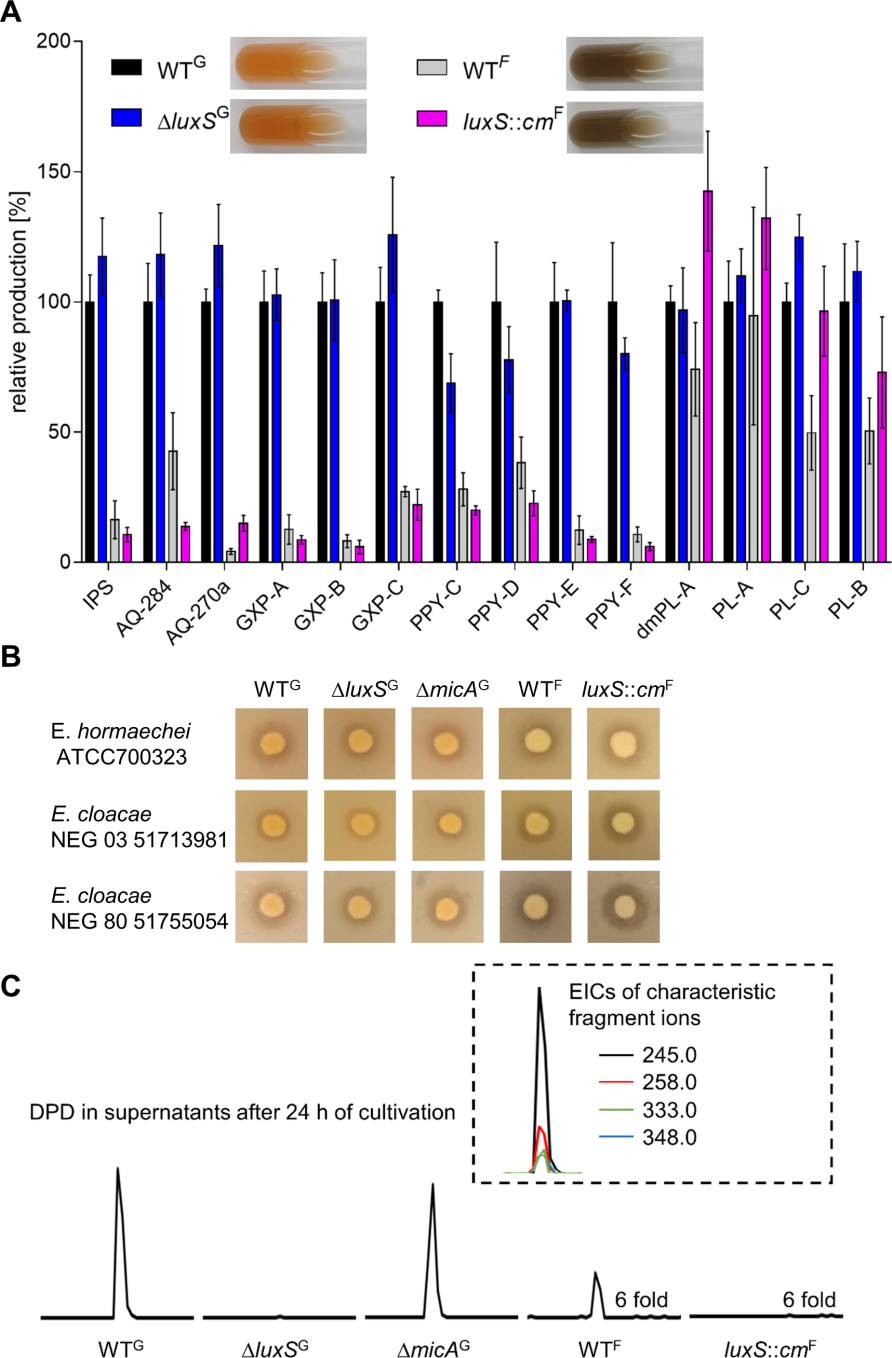
Comparison of NP production and AI-2 precursor levels (DPD) of *P. luminescens*^**G**^ and *P. luminescens*^**F**^. (A) Quantification of NP levels. Production was normalized with the OD_600_ when EE extracts were prepared and is given relative to *P. luminescens*^G^ WT production. Experiments were performed in quadruplicates. Pictures of the cultures were taken after 48 h of cultivation in LB broth at 30 °C. (B) Agar plate overlay assay for detection of carbapenem like antibiotic activity. (C) Detection of the AI-2 precursor DPD in supernatants of all investigated *P. luminescens* strains. In the dashed box all characteristic fragments of DPD detectable by GC-MS are shown all characteristic fragments of DPD detectable by GC-MS. Comparison of the strains is presented exemplary with the most abundant fragment. For better readability the scale of the chromatograms of WT^F^ and *luxS:: cm*^F^ were increased 6 fold. For cultivation conditions, extraction protocol, HPLC-MS measurement, quantification, overlay assay and AI-2 detection protocol see ‘Material and Methods’ section.

Global NP production of the *P. luminescens* strain used in our laboratory was compared with the original set of strains described by Evelyne Krin and colleagues (2006), who kindly provided the strains. In order to differentiate between these two sets of strains, they are referred to as *P. luminescens* Δ*luxS*^**G**^ (**G**ermany) and *P. luminescens luxS*::*cm*^**F**^(**F**rance), using the same superscripted letters for the corresponding *P. luminescens* WTs as well. Visual inspection of colony morphology and pigmentation of liquid culture and colonies revealed that *P. luminescens*^G^ and Δ*luxS*^G^ have a stronger red pigmentation in comparison to the other strain pair (Fig. 3) and therefore general differences in the NP production were analyzed. Here, a slightly different protocol than explained above was used. The strains were cultivated without XAD in LB medium and the cultures were extracted with EE. XAD (which binds NPs from the culture supernatants and can therefore slightly enhance production) was not used to detect also minor regulatory changes. Since there were still no detectable changes in NP production between *P. luminescens*^G^ and Δ*luxS*^G^ despite the different extraction method (Fig. 3) the strains from France and Germany were compared. *P. luminescens*^F^ produces noticeably lower amounts of IPS, AQs, GXPs and PPYs than *P. luminescens*^G^ reaching from 4 ± 1% (AQ-270a) to 43  ± 15% (AQ-284) of WT^G^ production. Only the phurealipids were produced in similar amounts (50 ± 14% to 95 ± 42%) by WT^F^. When comparing the NP production of *P. luminescens*^F^ with *luxS*::*cm*^F^, minor changes can be seen for AQ production. The mutant strain has a slightly impaired AQ-284 production, but at the same time produces more AQ-270a. For *luxS*::*cm*^F^ a higher amount of dmPL-A and PL-C than in the WT^F^ was detected, but no differences in PL production were obvious when comparing *P. luminescens* Δ*luxS*^*G*^ with WT^G^. HPLC-MS analysis of carbapenem production is very difficult due to known compound instability (Bonfiglio, Russo & Nicoletti, 2002) and therefore carbapenem production was monitored using an agar plate assay (Fig. 3B). In the presence of the antibiotic the growth of carbapenem sensitive *Enterobacter* strains is inhibited close to the *P. luminescens* colonies. With the three different *Enterobacter* strains used in this study no significant differences in the size of the inhibitions zones were detectable, when comparing TT01 WT^G^ with Δ*luxS*^G^ or TT01^F^ with *luxS*::*cm*^F^. But both WT strains had additional to the clear inhibition zone a diffuse inhibition zone, which was not visible for the respective *luxS* deficient strain when overlaid with *E. cloacae* NEG 80 51755054. The inhibitions zones for TT01^F^ were slightly bigger than for TT01^G^ when overlaid with both clinically isolated *E. cloacae* strains.

In order to check if deletion of *luxS* had an effect in all *P. luminescens* strains to the same extend when it comes to metabolite production, we quantified the produced amount of the AI-2 precursor DPD in the supernatant of 24 h old cultures. As expected, in supernatants of Δ*luxS*^*G*^ and *luxS*::*cm*^F^ no DPD was detectable, whereas in the respective WTs the precursor of AI-2 could be measured (Fig. 3C).

### Comparison of the genotype of the different *P. luminescens* mutant strains

**Figure 4:**
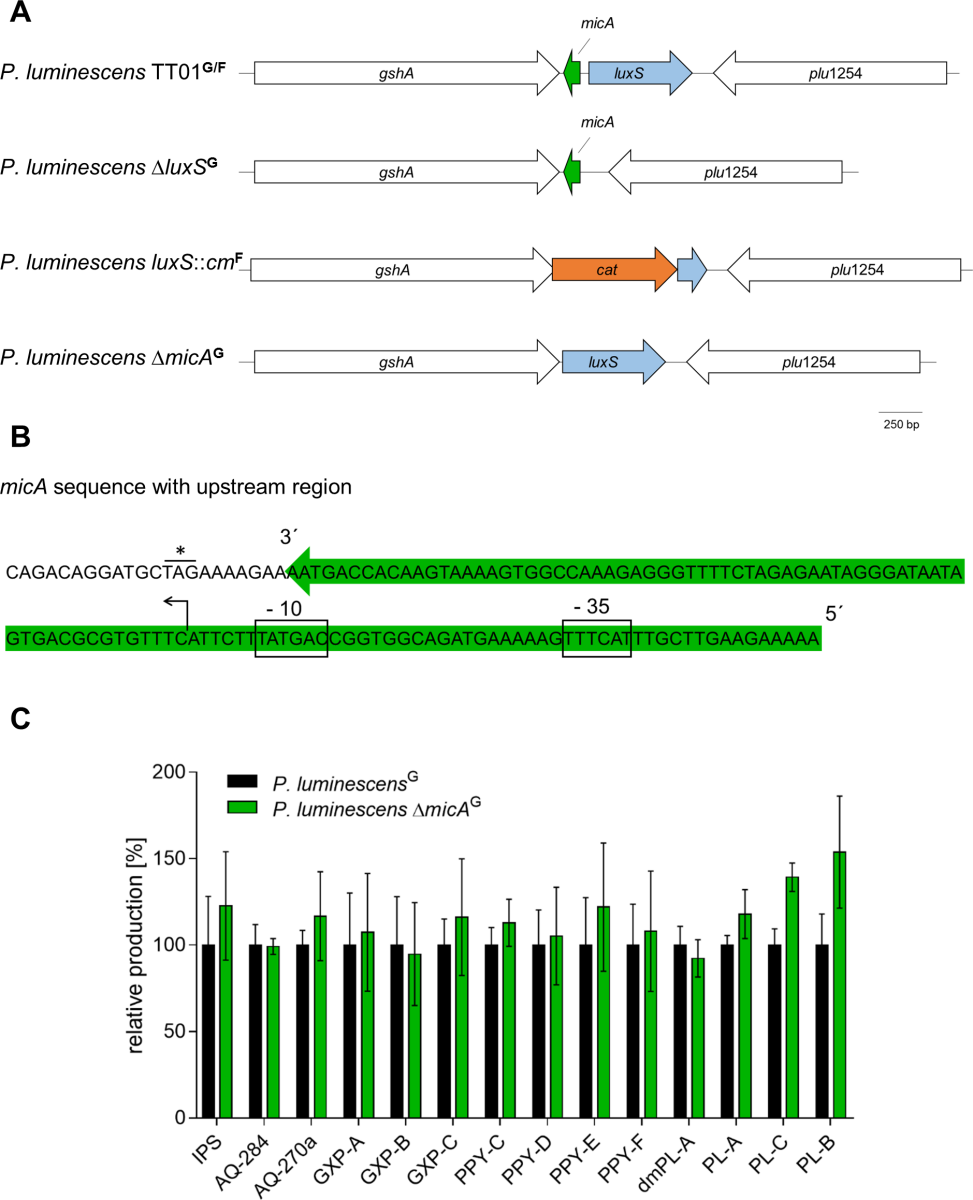
The small regulatory RNA *micA* is encoded upstream of *luxS* and deletion of *micA* does not influence NP production. (A) Comparison of the genotype of *P. luminescens*^G∕F^ and the corresponding mutant strains. In the WT, the *luxS* gene (light blue) is located between *gshA* and *plu1254*. Present between *luxS* and *gshA* is the small regulatory RNA *micA* (green). *cat* (orange): chloramphenicol resistance cassette. (B) *micA* sequence of *P. luminescens* TT01 with upstream region (marked in green) as described previously (Papamichail & Delihas, 2006). The sequence start is marked by an arrow, −10 and −35 regions are boxed. The stop codon of *gshA* is marked by a star. (C) NP production of *P. luminescens*^G^ and Δ*micA*^G^. Details on cultivation, extract preparation, HPLC-MS measurements and NP quantification see ‘Material and Methods’ section.

Since the *P. luminescens* strain pairs showed different levels of NPs, we compared the genotypes of Δ*luxS*^G^ and *luxS*::*cm*^F^ (Fig. 4A). The *luxS* gene is located between *gshA* encoding a γ-L-glutamyl-L-cysteine synthetase and *plu1254,* which encodes a protein with unknown function (Apontoweil & Berends, 1975). While *P. luminescens* Δ*luxS*^G^ is a deletion of the entire *luxS* gene including 13 bp of the upstream region of the CDS, *P. luminescens luxS*::*cm*^F^ has the *luxS* region replaced by a chloramphenicol resistance cassette (Derzelle et al., 2002). A detailed analysis showed that apart from the 5′ end of *luxS* and its complete upstream region, the last 37 bp of *gshA* including the stop codon are also deleted in this strain. A 200 bp fragment of the 3′ end of *luxS* was left in the genome. In *P. luminescens* and other enterobacteria the small non-coding RNA, *micA,* is located between *gshA* and *luxS* (Papamichail & Delihas, 2006; Vogel & Papenfort, 2006). In *P. luminescens* Δ*luxS*^G^ *micA* is still intact, while in *P. luminescens luxS*::*cm*^F^ it is deleted. To exclude that loss of *micA* was not responsible for the observed effects, we constructed the strain *P. luminescens* Δ*micA*^G^, in which *micA* together with its upstream region is deleted. Figure 4B shows the sequence of *micA* with upstream region as described previously (Papamichail & Delihas, 2006).

### *MicA* does not influence NP production in *P. luminescens*^*G*^

When the NP production of Δ*micA*^G^ and WT^G^ were compared no changes in compound levels were detectable (Fig. 4C). This was also true for carbapenem production monitored via an agar plate assay (Fig. 3B).

Since the somehow contradicting results observed for *P. luminescens* Δ*luxS*^G^ and *P. luminescens luxS*::*cm*^F^ cannot easily be explained by the presence or absence of *micA* and phenotypic differences as the pigmentation between WT^G^ and WT^F^ were obvious, the strains were compared by a number of assays addressing bioluminescence, resistance against oxidative stress, biofilm formation and virulence.

### Bioluminescence

**Figure 5:**
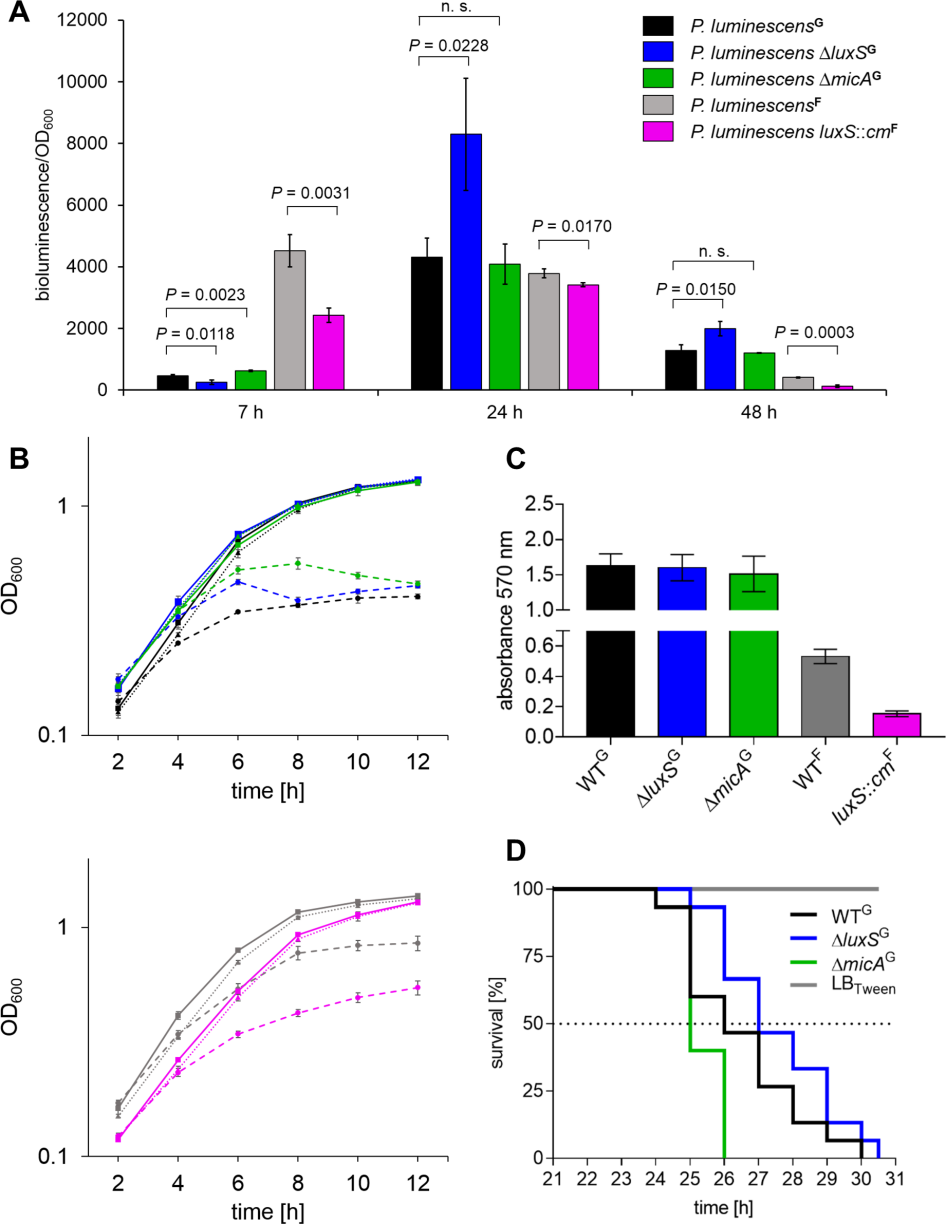
Phenotypic comparison of different ***P. luminescens*** strains. (A) Measured bioluminescence normalized by the OD_600_ of WT^G^, ΔluxS^G^, Δ*micA*^*G*^, WT^F^ and *luxS*::*cm*^*F*^ after 7, 24 and 48 h. (B) Growth curves (log scale) of all *P. luminescens* strains dependent from the treatment with H_2_O_2_ or paraquat. After 2 h of growth from OD_600_ = 0.25 reactants were added. Untreated: solid line/square, 10 mM H_2_O_2_: dotted line/triangle, 0.2 mM paraquat: dashed line/circle. (C) Biofilm formation on a polystyrene surface was compared after 72 h of cultivation via photometric quantification of biofilm bound dye upon crystal violet staining. (D) Comparison of virulence of WT^G^, Δ*luxS*^G^ and Δ*micA*^G^. Kaplan–Meier curve of 15 *G. mellonella* larvae infected per strain. The dashed line indicates the LT_50_ value. For details of all assays and bacterial cultivation see ‘Material and Methods’.

The name *Photorhabdus luminescens* comes from its ability to produce light via a luciferase dependent reaction (Winson et al., 1998). In *Vibrionaceae* bioluminescence production is controlled by AI-2 and the LuxPQUOR system (Rezzonico, Smits & Duffy, 2012). Bioluminescence measurements with all *P. luminescens* strains were performed confirming the previous results as bioluminescence of WT^F^ was two times higher than that of *luxS*::*cm*^F^ after 7 h during the exponential growth phase (Fig. 5A) (Krin et al., 2006). Interestingly, the overall bioluminescence of WT^G^ and the corresponding deletion strains was much lower at that time point, but here Δ*luxS*^G^ also showed a two-fold lower light production than WT^G^. After 24 h, all strains had reached the same bioluminescence level and there was no difference between WT^F^ and *luxS*::*cm*^F^. Remarkably, the bioluminescence of Δ*luxS*^G^ was now two-fold higher than that of WT^G^. Δ*micA*^G^ behaved very similarly to the WT with respect to bioluminescence indicating that the observed effects can be attributed to the loss of *luxS*.

### Oxidative stress assay

Oxidative stress assays were performed in order to test whether WT^G^ and Δ*luxS*^G^ or Δ*micA*^G^ behave differently. There are two different forms of oxidative stress that one can induce on bacteria - peroxide stress and superoxide stress (Farr & Kogoma, 1991). The oxidative defense response of each is distinct and involves different sets of proteins (Storz et al., 1990). Both pathways were tested with H_2_O_2_ used as an inducer of peroxide stress and paraquat as an inducer of superoxide stress. The exposure to 10 mM H_2_O_2_ did not show a significant effect on growth for any of the strains (Fig. 5B). This is in contrast to the previous results, where concentrations of 0.5 and 1 mM H_2_O_2_ already resulted in decreased growth of the WT^F^ (Krin et al., 2006). When WT^F^ and *luxS*::*cm*^F^ were tested in the same assay conditions as our strains (Fig. 5B) we could not observe a growth defect upon addition of the higher concentration of H_2_O_2_. The addition of 0.2 mM paraquat led to a reduced growth in all strains. WT^G^, Δ*luxS*^G^ and Δ*micA*^G^ were all influenced at the same level, so none of the deletions had an effect on paraquat sensitivity. However, *luxS*::*cm*^F^ showed an approximately two-fold lower final optical density than WT^F^ (0.54–0.85) compared to the untreated control.

### Biofilm formation

An assay monitoring the ability to produce biofilms on polystyrene surfaces was performed. For that, biofilm bound crystal violet was solubilized and quantified by measuring its absorbance at 570 nm. No changes in biofilm formation were detected between Δ*micA*^G^ or Δ*luxS*^G^ compared to WT^G^ (Fig. 5C). The biofilm formation of *P. luminescens*^G^ was around 3 times higher than that of *P. luminescens*^F^. In line with previous results (Krin et al., 2006) the *luxS*::*cm*^F^ strain was impaired in biofilm formation and produced 3.5 times less biofilm than the corresponding WT^F^.

### Virulence

Apart from its ability to produce a great variety of different small molecule NPs, *P. luminescens* is known for its capacity to kill a broad range of different insect larvae within one to two days using protein toxins (Bowen & Ensign, 1998). A virulence assay based on the infection of *G. mellonella* larvae with a low cell number of bacteria was performed and the LT_50_ values were calculated and compared. Since the LT_50_ values of the three strains (WT^G^ (LT_50_ = 26 h), Δ*luxS*^G^ (LT_50_ = 27 h) and Δ*micA*^G^ (LT_50_ = 25 h)) differed only by one hour and all strains killed infected insect larvae in less than 31 h, there are no significant changes in timing of the killing or in mortality rate of the insects (Fig. 5D).

## Discussion

We aimed to examine the global role of the regulatory protein LuxS on NP production that originally was described to influence carbapenem production (Derzelle et al., 2002). However, no differences in NP production were observed showing that LuxS does not globally affect NP production levels in *P. luminescens*^G^, *X. nematophila* or *X. szentirmaii*. The investigated compounds are derived from several different biosynthetic pathways including NRPS (GXPs, RXPs, MVAP, NMT, XNCs, XTP, XABs, SZT, XP), polyketide synthases (PKS) (AQs, IPS, PPYs, XNCs), NPs derived from intermediates of fatty acid biosynthesis (PLs) or other biosynthetic pathways (XF-A) (for detailed mechanisms see references in Table 4). Altered AQ production in *luxS*::*cm*^F^cannot be directly linked to a LuxS dependent regulation. Both AQ-270a and AQ-284 are derived via different methylations from AQ-256, which is produced by the enzyme machinery encoded by the *antA-I* cluster. AQ-284 possesses one additional methyl group compared to AQ-270a (Brachmann et al., 2007). Thus, changes in the ratio of AQ-270a to AQ-284 may reflect impairments in the methylation pathway, due to the loss of LuxS in the AMC. Summing up these results we conclude that LuxS is not a global regulator of NP production in either *Photorhabdus* or *Xenorhabdus*.

A global analysis for the presence of the *lsr* operon, whose gene products are responsible for AI-2 uptake, was also performed. It is assumed that only when a bacterium has the complete set of *lsr* genes, it can use AI-2 as a real QS molecule (Rezzonico & Duffy, 2008; Brito et al., 2013). When comparing 25 *Xenorhabdus* and *Photorhabdus* strains with respect to the occurrence of the genes of the *lsr* operon, no pattern that follows the phylogeny was observable (Fig. 2A). This goes in line with an earlier study assigning *lsr* genes to be important for bacteria-nematode interaction (Gaudriault et al., 2006). There it was concluded that the *lsr* locus was present in a *Photorhabdus*/*Xenorhabdus* ancestor and was individually lost in different strains lost during evolution in different strains that are not living in symbiosis with *H. bacteriophora*. Presence of the entire operon in different *Xenorhabdus* strains indicates that *H. bacteriophora* symbiosis is not the only pressure to keep the cluster. However, the strains not harboring the complete operon still encode the kinase LsrK and the AI-2 degrading enzymes LsrFG. Investigations of different components of the Lsr transporter in *S. typhimurium* and *E. coli* revealed that deletions of *lsrB* (Taga, Miller & Bassler, 2003) or *lsrCDB* (Xavier & Bassler, 2005) still led to a slow uptake of AI-2. Only deletion of the kinase *lsrK* stopped the internalization of AI-2 from the supernatant completely. It was concluded that AI-2 can fulfill its function only in a phosphorylated state and that additional uptake mechanisms for AI-2 must exist. Since all analyzed strains still carry *lsrK* it is possible that these strains are still able to take up AI-2 via alternative receptors and then process the signal. The ribose-binding protein RbsB was shown to interact with AI-2 in *Actinobacillus actinomycetemcomitans* (James et al., 2006) and *Haemophilus influenza* (Armbruster et al., 2011). In *X. szentirmaii rbsB* is present in the genome, while in *X. nematophila* it is not. Additionally, the phosphoenolpyruvate phosphotransferase system (PTS), which is found in both *Xenorhabdus* strains, was shown to be involved in the initial uptake of AI-2 (Pereira et al., 2012). In summary, one can postulate that uptake of AI-2 is not the limiting factor and if there were effects on NP production, we would have seen it in *P. luminescens*, *X. nematophila* and *X. szentirmaii*, upon *luxS* deletion.

Although this study concentrated on the influence of the possible global regulator LuxS on three selected strains, we could not neglect the fact that “our” *P. luminescens* TT01 WT behaved differently from what was described in the literature (Krin et al., 2006). A number of phenotypic differences between *P. luminescens*^G^ and *P. luminescens*^F^ have been uncovered including different behavior in bioluminescence production. How bioluminescence is activated in *P. luminescens* is still unclear. What can be noted, is that WT^G^ and WT^F^ show a different time-dependent development of bioluminescence. In the transcriptome analyses Krin et al. (2006) observed that the expression of the *luxCDABE* genes was not altered in the *luxS* mutant strain and justified the change in bioluminescence by altered concentrations of spermidine in the cells. Spermidine can react with aldehydes, the substrate for the bioluminescence reaction, and quench the light development by scavenging the substrate. The role of spermidine in this process was concluded from reduced expression of several genes encoding proteins related to polyamine metabolism. Interestingly, *luxS*::*cm*^F^ showed slightly enhanced phurealipid (PL) production (dmPL-A and PL-C). Upregulation of all PLs also could be seen in Δ*luxS*^G^ in an additional analysis. PL biosynthesis also starts with fatty acid-derived aldehydes (Nollmann et al., 2015).

One biosynthesis gene cluster with reduced expression in the *luxS*::*cm*^F^ mutant (*plu4563*-*plu4568*) was later shown to be responsible for the production of the *Photorhabdus* clumping factor Pcf (Brachmann et al., 2013; Brameyer et al., 2015). Therefore, it is not surprising that biofilm formation was affected in the *luxS*::*cm*^F^ mutant (Krin et al., 2006). We were not able to observe impaired biofilm formation for the Δ*luxS*^G^ mutant, although we saw the described reduction of biofilm formation for *luxS*::*cm*^F^ in the performed microtiter plate assay (Fig. 5C). However, confusing and often contradicting results when analyzing the role of LuxS in biofilm formation have been described before (for summary see review (Hardie & Heurlier, 2008)).

In a computational approach analyzing the genome of *P. luminescens,* the small RNA *micA,* often assigned to regulation of the outer membrane protein OmpA (Udekwu et al., 2005; Rasmussen et al., 2005; Johansen et al., 2006), was predicted to be encoded upstream of *luxS* (Papamichail & Delihas, 2006). In *Salmonella* it was uncovered that impaired biofilm formation correlated with *luxS* deletion was indeed caused by impaired *micA* expression upon *luxS* deletion destroying the putative promoter region of *micA* (Kint et al., 2010). No such effect was observed for the Δ*micA*^G^ strain. The deletion of *micA* did not impair *luxS* expression, since the AI-2 precursor was measured in comparable amounts in WT^G^ and in Δ*micA*^G^, whereas it was not detectable in the supernatant of Δ*luxS*^G^ (Fig. 3C).

Additionally, no influence of either LuxS or *micA* on superoxide and hydrogen peroxide stress was obvious besides the differences between the F and G strain (pairs). When compared to other studies investigating the effect of *luxS* mutations on the oxidative stress response, no consistent results exist that describe how AI-2 directly influences expression of genes involved in the oxidative stress defense. Studies with *Streptococcus mutans* (Wen & Burne, 2004) and *Porphyromonas gingivalis* (Yuan, Hillman & Progulske-Fox, 2005) showed that the corresponding *luxS* mutants had a higher tolerance towards H_2_O_2_. Contrary results were obtained for *Campylobacter jejuni* (He et al., 2008) and *Yersinia pestis* (Yu et al., 2013), where the *luxS* mutant strains were impaired in their resistance to oxidative stress. There exists also a study by Wilson et al. where they developed a model for *Lactobacillus reuteri*, which explains the altered expression (monitored with a microarray analysis) of redox stress involved genes by the metabolic role of LuxS (Wilson et al., 2012).

In a previously performed killing assay, a delay in killing larvae of the african cotton leafworm; *Spodoptera littoralis* (Krin et al., 2006) was observed, whereas in this study neither a difference in mortality, nor a difference in the timing of the killing of *G. mellonella* infected with the Δ*luxS* strain was obvious. Conflicting results upon *luxS* deletions in different species of the same genus have been reported before. Coulthurst et al. presented non-uniform phenotypes, including differences in NP production and virulence in *luxS* deficient mutants of two *Serratia* species and concluded that the regulatory effects of LuxS depend on the strain (Coulthurst, Kurz & Salmond, 2004). Barnad et al. summed up the phenotypes of *luxS* mutants in different *Erwinia* strains and differences in LuxS mediated regulation of virulence let them also conclude that LuxS seems to have different functions in different strains of *Erwinia* (Barnard & Salmond, 2007; Coulthurst, Lilley & Salmond, 2006; Laasik, Andresen & Mae, 2006). Furthermore, it must be kept in mind that some phenotypic assays which were described before with *luxS*::*cm*^F^ and the ones which were performed in this study were conducted following slightly different protocols. One important variation is that Krin et al. have grown their strains in presence of 10 µM sodium borate since AI-2 is either a borated or non-borated molecule (Miller et al., 2004; Chen et al., 2002) and in order to avoid any shortage borate was added to the media. Like in *S. typhimurium* the *lsr* operon is made responsible for the transmission of AI-2 into the cells of *P. luminescens.* LsrB from *S. typhimurium* was shown to bind a non-borated version of AI-2 and even an inhibition of AI-2 mediated signaling was shown after addition of boric acid (Miller et al., 2004). Therefore, all experiments in this study were performed without addition of boric acid but using normal glassware.

## Conclusion

In summary, we show that LuxS is not involved in the global regulation of NPs in *P. luminescens*, *X. nematophila* and *X. szentirmaii* as analyzed by HPLC-MS analysis and NP quantification. The overlay assay for the detection of carbapenem like antibiotics did not indicate any significant differences in antibiotic production of the WT and the respective mutant strains, when comparing the results seen for three different *Enterobacter* strains. Additionally, the known regulatory RNA *micA* does not influence NP production in *P. luminescens*.

Another result of the comparison of the different F and G strains is that these strains most likely have evolved independently in the different laboratories and therefore show very different phenotypes. Similar to our observations concerning the role of LuxS in *P. luminescens*^G^ and *P. luminescens*^F^, divergent experimental outcomes for the role of Lrp in regulation of IPS biosynthesis have been explained with genomic changes in the strains used by two different groups (Kontnik, Crawford & Clardy, 2010; Lango-Scholey et al., 2013). Genome sequencing of *Photorhabdus luminescens* TT01 revealed that phage remnants make up already 4% of the entire genome. Additional to that, 195 IS/IS fragments and 711 ERIC (enterobacterial repetitive intergenic consensus) sequences have been found (Hulton, Higgins & Sharp, 1991; Duchaud et al., 2003). The huge amount of mobile genetic elements underscores the idea of a very flexible genome with rearrangements occurring often (Duchaud et al., 2003). Since in most laboratories (including ours) entomopathogenic bacteria are usually not grown with their host nematode, such changes might occur quickly as is also shown recently in experimental evolution experiments (Morran et al., 2016). The effects of inter-laboratory evolution were also revealed by a comparative analysis of nine laboratory “wild type” strains of the model organism *Myxococcus xanthus* DK1622 (Bradley et al., 2016).

## Additional Information and Declarations

### Data Availability

The following information was supplied regarding data availability: Genomes are uploaded at Genbank. The accession numbers are as follows: Photorhabdus asymbiotica (NC_012962.1) Photorhabdus temperata subsp. thracensis (NZ_CP011104.1) Photorhabdus luminescens TT01 (NC_005126.1) Photorhabdus luminescens PB45.5 (LOIC00000000) Xenorhabdus cabanillasii JM26 (GCA_000531755.1) Xenorhabdus bovienii SS-2004 (NC_013892.1) Xenorhabdus hominickii DSM 17903 (NZ_CP016176.1) Xenorhabdus nematophila ATCC 19061 (NC_014228.1) Xenorhabdus szentirmaii DSM 16338 (GCA_000531455.1) Xenorhabdus mauleonii DSM 17908 (GCA_900113945.1) Xenorhabdus bedingii DSM 4764 (MUBK00000000) Xenorhabdus vietnamensis DSM 22392 (MUBJ00000000) Xenorhabdus doucetiae FRM16 (GCA_000968195.1) Xenorhabdus sp. 30TX1 (MKGR00000000) Xenorhabdus sp. DL20 (MKGQ00000000).

## References

[ref-1] Apontoweil P, Berends W (1975). Mapping of *gshA*, a gene for the biosynthesis of glutathione in *Eschericha coli* K12. Molecular Genetics and Genomics.

[ref-2] Armbruster CE, Pang B, Murrah K, Juneau RA, Perez AC, Weimer KED, Swords WE (2011). RbsB (NTHI_0632) mediates quorum signal uptake in nontypeable *Haemophilus influenzae* strain 86-028NP. Molecular Microbiology.

[ref-3] Barnard AML, Salmond GPC (2007). Quorum sensing in *Erwinia* species. Analytical and Bioanalytical Chemistry.

[ref-4] Bennett HPJ, Clarke DJ (2005). The *pbgPE* operon in *Photorhabdus luminescens* is required for pathogenicity and symbiosis. Journal of Bacteriology.

[ref-5] Bode HB (2009). Entomopathogenic bacteria as a source of secondary metabolites. Current Opinion in Chemical Biology.

[ref-6] Bode HB, Reimer D, Fuchs SW, Kirchner F, Dauth C, Kegler C, Lorenzen W, Brachmann AO, Grün P (2012). Determination of the absolute configuration of peptide natural products by using stable isotope labeling and mass spectrometry. Chemistry.

[ref-7] Bonfiglio G, Russo G, Nicoletti G (2002). Recent developments in carbapenems. Expert Opinion on Investigational Drugs.

[ref-8] Bowen DJ, Ensign JC (1998). Purification and characterization of a high-molecular-weight insecticidal protein complex produced by the entomopathogenic bacterium *Photorhabdus luminescens*. Applied and Environmental Microbiology.

[ref-9] Brachmann AO, Brameyer S, Kresovic D, Hitkova I, Kopp Y, Manske C, Schubert K, Bode HB, Heermann R (2013). Pyrones as bacterial signaling molecules. Nature Chemical Biology.

[ref-10] Brachmann AO, Forst S, Furgani GM, Fodor A, Bode HB (2006). Xenofuranones A and B: phenylpyruvate dimers from *Xenorhabdus szentirmaii*. Journal of Natural Products.

[ref-11] Brachmann AO, Joyce SA, Jenke-Kodama H, Schwar G, Clarke DJ, Bode HB (2007). A type II polyketide synthase is responsible for anthraquinone biosynthesis in *Photorhabdus luminescens*. Chembiochem.

[ref-12] Bradley MD, Neu D, Bahar F, Welch RD (2016). Inter-laboratory evolution of a model organism and its epistatic effects on mutagenesis screens. Scientific Reports.

[ref-13] Brameyer S, Kresovic D, Bode HB, Heermann R (2014). LuxR solos in *Photorhabdus* species. Frontiers in Cellular and Infection Microbiology.

[ref-14] Brameyer S, Kresovic D, Bode HB, Heermann R (2015). Dialkylresorcinols as bacterial signaling molecules. Proceedings of the National Academy of Sciences of the United States of America.

[ref-15] Brito PH, Rocha EPC, Xavier KB, Gordo I (2013). Natural genome diversity of AI-2 quorum sensing in *Escherichia coli*: conserved signal production but labile signal reception. Genome Biology and Evolution.

[ref-16] Cai X, Challinor VL, Zhao L, Reimer D, Adihou H, Grün P, Kaiser M, Bode HB (2017a). Biosynthesis of the antibiotic nematophin and its elongated derivatives in entomopathogenic bacteria. Organic Letters.

[ref-17] Cai X, Nowak S, Wesche F, Bischoff I, Kaiser M, Fürst R, Bode HB (2017b). Entomopathogenic bacteria use multiple mechanisms for bioactive peptide library design. Nature Chemistry.

[ref-18] Chen X, Schauder S, Potier N, Van Dorsselaer A, Pelczer I, Bassler BL, Hughson FM (2002). Structural identification of a bacterial quorum-sensing signal containing boron. Nature.

[ref-19] Coulthurst SJ, Kurz CL, Salmond GPC (2004). *luxS* mutants of *Serratia* defective in autoinducer-2-dependent ‘quorum sensing’ show strain-dependent impacts on virulence and production of carbapenem and prodigiosin. Microbiology.

[ref-20] Coulthurst SJ, Lilley KS, Salmond GPC (2006). Genetic and proteomic analysis of the role of *luxS* in the enteric phytopathogen, *Erwinia carotovora*. Molecular Plant Pathology.

[ref-21] Derzelle S, Duchaud E, Kunst F, Danchin A, Bertin P (2002). Identification, characterization, and regulation of a cluster of genes involved in carbapenem biosynthesis in *Photorhabdus luminescens*. Applied and Environmental Microbiology.

[ref-22] Duchaud E, Rusniok C, Frangeul L, Buchrieser C, Givaudan A, Taourit S, Bocs S, Boursaux-Eude C, Chandler M, Charles J-F, Dassa E, Derose R, Derzelle S, Freyssinet G, Gaudriault S, Medigue C, Lanois A, Powell K, Siguier P, Vincent R, Wingate V, Zouine M, Glaser P, Boemare N, Danchin A, Kunst F (2003). The genome sequence of the entomopathogenic bacterium *Photorhabdus luminescens*. Nature Biotechnology.

[ref-23] Durfee T, Nelson R, Baldwin S, Plunkett G, Burland V, Mau B, Petrosino JF, Qin X, Muzny DM, Ayele M, Gibbs RA, Csorgo B, Posfai G, Weinstock GM, Blattner FR (2008). The complete genome sequence of *Escherichia coli* DH10B: insights into the biology of a laboratory workhorse. Journal of Bacteriology.

[ref-24] Engel Y, Windhorst C, Lu X, Goodrich-Blair H, Bode HB (2017). The global regulators Lrp, LeuO, and HexA control secondary metabolism in entomopathogenic bacteria. Frontiers in Microbiology.

[ref-25] Farr SB, Kogoma T (1991). Oxidative stress responses in *Escherichia coli* and *Salmonella typhimurium*. Microbiological Reviews.

[ref-26] Fischer-Le Saux M, Viallard V, Brunel B, Normand P, Boemare NE (1999). Polyphasic classification of the genus *Photorhabdus* and proposal of new taxa: *P. luminescens* subsp. *luminescens* subsp. nov., *P. luminescens* subsp. *akhurstii* subsp. nov., *P. luminescens* subsp. *laumondii* subsp. nov. *P. temperata* sp. nov., *P. temperata* subsp. *temperata* subsp. nov. and *P. asymbiotica* sp. nov.. International Journal of Systematic Bacteriology.

[ref-27] Gaudriault S, Duchaud E, Lanois A, Canoy A-S, Bourot S, Derose R, Kunst F, Boemare N, Givaudan A (2006). Whole-genome comparison between *Photorhabdus* strains to identify genomic regions involved in the specificity of nematode interaction. Journal of Bacteriology.

[ref-28] Goodrich-Blair H, Clarke DJ (2007). Mutualism and pathogenesis in *Xenorhabdus* and *Photorhabdus*: two roads to the same destination. Molecular Microbiology.

[ref-29] Grant SG, Jessee J, Bloom FR, Hanahan D (1990). Differential plasmid rescue from transgenic mouse DNAs into *Escherichia coli* methylation-restriction mutants. Proceedings of the National Academy of Sciences of the United States of America.

[ref-30] Gualtieri M, Ogier J-C, Pages S, Givaudan A, Gaudriault S (2014). Draft genome sequence and annotation of the entomopathogenic bacterium *Xenorhabdus szentirmaii* strain DSM16338. Genome Announcements.

[ref-31] Hardie KR, Heurlier K (2008). Establishing bacterial communities by ‘word of mouth’: LuxS and autoinducer 2 in biofilm development. Nature Reviews. Microbiology.

[ref-32] He Y, Frye JG, Strobaugh TP, Chen C-Y (2008). Analysis of AI-2/LuxS-dependent transcription in *Campylobacter jejuni* strain 81-176. Foodborne Pathogens and Disease.

[ref-33] Heinrich AK, Glaeser A, Tobias NJ, Heermann R, Bode HB (2016). Heterogeneous regulation of bacterial natural product biosynthesis via a novel transcription factor. Heliyon.

[ref-34] Hulton CS, Higgins CF, Sharp PM (1991). ERIC sequences: a novel family of repetitive elements in the genomes of *Escherichia coli*, *Salmonella typhimurium* and other enterobacteria. Molecular Microbiology.

[ref-35] James D, Shao H, Lamont RJ, Demuth DR (2006). The *Actinobacillus actinomycetemcomitans* ribose binding protein RbsB interacts with cognate and heterologous autoinducer 2 signals. Infection and Immunity.

[ref-36] Johansen J, Rasmussen AA, Overgaard M, Valentin-Hansen P (2006). Conserved small non-coding RNAs that belong to the sigmaE regulon: role in down-regulation of outer membrane proteins. Journal of Molecular Biology.

[ref-37] Joyce SA, Brachmann AO, Glazer I, Lango L, Schwär G, Clarke DJ, Bode HB (2008). Bacterial biosynthesis of a multipotent stilbene. Angewandte Chemie (International Ed. in English).

[ref-38] Kegler C, Nollmann FI, Ahrendt T, Fleischhacker F, Bode E, Bode HB (2014). Rapid determination of the amino acid configuration of xenotetrapeptide. Chembiochem.

[ref-39] Kint G, De Coster D, Marchal K, Vanderleyden J, De Keersmaecker SCJ (2010). The small regulatory RNA molecule MicA is involved in *Salmonella enterica* serovar Typhimurium biofilm formation. BMC Microbiology.

[ref-40] Kontnik R, Crawford JM, Clardy J (2010). Exploiting a global regulator for small molecule discovery in *Photorhabdus luminescens*. ACS Chemical Biology.

[ref-41] Krin E, Chakroun N, Turlin E, Givaudan A, Gaboriau F, Bonne I, Rousselle J-C, Frangeul L, Lacroix C, Hullo M-F, Marisa L, Danchin A, Derzelle S (2006). Pleiotropic role of quorum-sensing autoinducer 2 in *Photorhabdus luminescens*. Applied and Environmental Microbiology.

[ref-42] Laasik E, Andresen L, Mae A (2006). Type II quorum sensing regulates virulence in *Erwinia carotovora* ssp. carotovora. FEMS Microbiology Letters.

[ref-43] Lang G, Kalvelage T, Peters A, Wiese J, Imhoff JF (2008). Linear and cyclic peptides from the entomopathogenic bacterium *Xenorhabdus nematophilus*. Journal of Natural Products.

[ref-44] Lango-Scholey L, Brachmann AO, Bode HB, Clarke DJ (2013). The expression of *stlA* in *Photorhabdus luminescens* is controlled by nutrient limitation. PLOS ONE.

[ref-45] Li J, Chen G, Webster JM (1997). Nematophin, a novel antimicrobial substance produced by *Xenorhabdus nematophilus* (Enterobactereaceae). Canadian Journal of Microbiology.

[ref-46] Marques JC, Oh IK, Ly DC, Lamosa P, Ventura MR, Miller ST, Xavier KB (2014). LsrF, a coenzyme A-dependent thiolase, catalyzes the terminal step in processing the quorum sensing signal autoinducer-2. Proceedings of the National Academy of Sciences of the United States of America.

[ref-47] McInerney BV, Taylor WC, Lacey MJ, Akhurst RJ, Gregson RP (1991). Biologically active metabolites from *Xenorhabdus* spp., Part 2. Benzopyran-1-one derivatives with gastroprotective activity. Journal of Natural Products.

[ref-48] Merritt JH, Kadouri DE, O’Toole GA (2005). Growing and analyzing static biofilms. Current Protocols in Microbiology.

[ref-49] Miller MB, Bassler BL (2001). Quorum sensing in bacteria. Annual Reviews in Microbiology.

[ref-50] Miller ST, Xavier KB, Campagna SR, Taga ME, Semmelhack MF, Bassler BL, Hughson FM (2004). *Salmonella typhimurium* recognizes a chemically distinct form of the bacterial quorum-sensing signal AI-2. Molecular Cell.

[ref-51] Morran LT, Penley MJ, Byrd VS, Meyer AJ, O’Sullivan TS, Bashey F, Goodrich-Blair H, Lively CM (2016). Nematode-bacteria mutualism: selection within the mutualism supersedes selection outside of the mutualism. Evolution; International Journal of Organic Evolution.

[ref-52] Nollmann FI, Dowling A, Kaiser M, Deckmann K, Grosch S, ffrench-Constant R, Bode HB (2012). Synthesis of szentiamide, a depsipeptide from entomopathogenic *Xenorhabdus szentirmaii* with activity against *Plasmodium falciparum*. Beilstein Journal of Organic Chemistry.

[ref-53] Nollmann FI, Heinrich AK, Brachmann AO, Morisseau C, Mukherjee K, Casanova-Torres ÁM, Strobl F, Kleinhans D, Kinski S, Schultz K, Beeton ML, Kaiser M, Chu Y-Y, Phan Ke L, Thanwisai A, Bozhüyük KAJ, Chantratita N, Götz F, Waterfield NR, Vilcinskas A, Stelzer EHK, Goodrich-Blair H, Hammock BD, Bode HB (2015). A *Photorhabdus* natural product inhibits insect juvenile hormone epoxide hydrolase. Chembiochem.

[ref-54] Ohlendorf B, Simon S, Wiese J, Imhoff JF (2011). Szentiamide, an N-formylated cyclic depsipeptide from *Xenorhabdus szentirmaii* DSM 16338. Natural Product Communications.

[ref-55] Orchard SS, Goodrich-Blair H (2004). Identification and functional characterization of a *Xenorhabdus nematophila* oligopeptide permease. Applied and Environmental Microbiology.

[ref-56] Papamichail D, Delihas N (2006). Outer membrane protein genes and their small non-coding RNA regulator genes in *Photorhabdus luminescens*. Biology Direct.

[ref-57] Pereira CS, Santos AJM, Bejerano-Sagie M, Correia PB, Marques JC, Xavier KB (2012). Phosphoenolpyruvate phosphotransferase system regulates detection and processing of the quorum sensing signal autoinducer-2. Molecular Microbiology.

[ref-58] Pereira CS, Thompson JA, Xavier KB (2013). AI-2-mediated signalling in bacteria. FEMS Microbiology Reviews.

[ref-59] Philippe N, Alcaraz J-P, Coursange E, Geiselmann J, Schneider D (2004). Improvement of pCVD442, a suicide plasmid for gene allele exchange in bacteria. Plasmid.

[ref-60] Rasmussen AA, Eriksen M, Gilany K, Udesen C, Franch T, Petersen C, Valentin-Hansen P (2005). Regulation of *ompA* mRNA stability: the role of a small regulatory RNA in growth phase-dependent control. Molecular Microbiology.

[ref-61] Reimer D, Cowles KN, Proschak A, Nollmann FI, Dowling AJ, Kaiser M, ffrench-Constant R, Goodrich-Blair H, Bode HB (2013). Rhabdopeptides as insect-specific virulence factors from entomopathogenic bacteria. Chembiochem.

[ref-62] Reimer D, Luxenburger E, Brachmann AO, Bode HB (2009). A new type of pyrrolidine biosynthesis is involved in the late steps of xenocoumacin production in *Xenorhabdus nematophila*. Chembiochem.

[ref-63] Reimer D, Pos KM, Thines M, Grun P, Bode HB (2011). A natural prodrug activation mechanism in nonribosomal peptide synthesis. Nature Chemical Biology.

[ref-64] Rezzonico F, Duffy B (2008). Lack of genomic evidence of AI-2 receptors suggests a non-quorum sensing role for *luxS* in most bacteria. BMC Microbiology.

[ref-65] Rezzonico F, Smits THM, Duffy B (2012). Detection of AI-2 receptors in genomes of Enterobacteriaceae suggests a role of type-2 quorum sensing in closed ecosystems. Sensors.

[ref-66] Simon R, Priefer U, Pühler A (1983). A broad host range mobilization system for in vivo genetic engineering: transposon mutagenesis in gram negative bacteria. Nature Biotechnology.

[ref-67] Storz G, Tartaglia LA, Farr SB, Ames BN (1990). Bacterial defenses against oxidative stress. Trends in Genetics.

[ref-68] Taga ME, Miller ST, Bassler BL (2003). Lsr-mediated transport and processing of AI-2 in *Salmonella typhimurium*. Molecular Microbiology.

[ref-69] Thiel V, Vilchez R, Sztajer H, Wagner-Döbler I, Schulz S (2009). Identification, quantification, and determination of the absolute configuration of the bacterial quorum-sensing signal autoinducer-2 by gas chromatography-mass spectrometry. Chembiochem.

[ref-70] Thoma S, Schobert M (2009). An improved *Escherichia coli* donor strain for diparental mating. FEMS Microbiology Letters.

[ref-71] Tobias NJ, Heinrich AK, Eresmann H, Wright PR, Neubacher N, Backofen R, Bode HB (2017). *Photorhabdus*-nematode symbiosis is dependent on *hfq*-mediated regulation of secondary metabolites. Environmental Microbiology.

[ref-72] Udekwu KI, Darfeuille F, Vogel J, Reimegard J, Holmqvist E, Wagner EGH (2005). Hfq-dependent regulation of OmpA synthesis is mediated by an antisense RNA. Genes & Development.

[ref-73] Vogel J, Papenfort K (2006). Small non-coding RNAs and the bacterial outer membrane. Current Opinion in Microbiology.

[ref-74] Waters CM, Bassler BL (2005). Quorum sensing: cell-to-cell communication in bacteria. Annual Review of Cell and Developmental Biology.

[ref-75] Wen ZT, Burne RA (2004). LuxS-mediated signaling in *Streptococcus mutans* is involved in regulation of acid and oxidative stress tolerance and biofilm formation. Journal of Bacteriology.

[ref-76] Wilson CM, Aggio RBM, O’Toole PW, Villas-Boas S, Tannock GW (2012). Transcriptional and metabolomic consequences of LuxS inactivation reveal a metabolic rather than quorum-sensing role for LuxS in *Lactobacillus reuteri* 100-23. Journal of Bacteriology.

[ref-77] Winson MK, Swift S, Hill PJ, Sims CM, Griesmayr G, Bycroft BW, Williams P, Stewart GSAB (1998). Engineering the *lux* CDABE genes from *Photorhabdus luminescens* to provide a bioluminescent reporter for constitutive and promoter probe plasmids and mini-Tn5 constructs. FEMS Microbiology Letters.

[ref-78] Winzer K, Hardie KR, Burgess N, Doherty N, Kirke D, Holden MTG, Linforth R, Cornell KA, Taylor AJ, Hill PJ, Williams P (2002). LuxS: its role in central metabolism and the *in vitro* synthesis of 4-hydroxy-5-methyl-3(2H)-furanone. Microbiology.

[ref-79] Xavier KB, Bassler BL (2005). Regulation of uptake and processing of the quorum-sensing autoinducer AI-2 in *Escherichia coli*. Journal of Bacteriology.

[ref-80] Yu J, Madsen ML, Carruthers MD, Phillips GJ, Kavanaugh JS, Boyd JM, Horswill AR, Minion FC (2013). Analysis of autoinducer-2 quorum sensing in *Yersinia pestis*. Infection and Immunity.

[ref-81] Yuan L, Hillman JD, Progulske-Fox A (2005). Microarray analysis of quorum-sensing-regulated genes in *Porphyromonas gingivalis*. Infection and Immunity.

[ref-82] Zhou Q, Grundmann F, Kaiser M, Schiell M, Gaudriault S, Batzer A, Kurz M, Bode HB (2013). Structure and biosynthesis of xenoamicins from entomopathogenic *Xenorhabdus*. Chemistry.

